# A local average distance descriptor for flexible protein structure comparison

**DOI:** 10.1186/1471-2105-15-95

**Published:** 2014-04-02

**Authors:** Hsin-Wei Wang, Chia-Han Chu, Wen-Ching Wang, Tun-Wen Pai

**Affiliations:** 1 Department of Computer Science and Engineering, National Taiwan Ocean University, Keelung, Taiwan; 2 Biomedical Science and Engineering Center, National Tsing Hua University, Hsinchu, Taiwan; 3 Institute of Molecular and Cellular Biology and Department of Life Science, National Tsing Hua University, Hsinchu, Taiwan

## Abstract

**Background:**

Protein structures are flexible and often show conformational changes upon binding to other molecules to exert biological functions. As protein structures correlate with characteristic functions, structure comparison allows classification and prediction of proteins of undefined functions. However, most comparison methods treat proteins as rigid bodies and cannot retrieve similarities of proteins with large conformational changes effectively.

**Results:**

In this paper, we propose a novel descriptor, local average distance (LAD), based on either the geodesic distances (GDs) or Euclidean distances (EDs) for pairwise flexible protein structure comparison. The proposed method was compared with 7 structural alignment methods and 7 shape descriptors on two datasets comprising hinge bending motions from the MolMovDB, and the results have shown that our method outperformed all other methods regarding retrieving similar structures in terms of precision-recall curve, retrieval success rate, R-precision, mean average precision and F_1_-measure.

**Conclusions:**

Both ED- and GD-based LAD descriptors are effective to search deformed structures and overcome the problems of self-connection caused by a large bending motion. We have also demonstrated that the ED-based LAD is more robust than the GD-based descriptor. The proposed algorithm provides an alternative approach for blasting structure database, discovering previously unknown conformational relationships, and reorganizing protein structure classification.

## Background

Protein structure comparison plays an important role in predicting functions of novel proteins [1] and several methods have been developed for pairwise [2-8] and multiple [9-16] comparisons. Most existing methods of structure comparison treat proteins as rigid bodies; however, protein structures are flexible and conformationally changeable in response to binding another molecules relating with biological functions such as immune protection, enzymatic catalysis and cellular locomotion [17,18]. Such structural variations caused rigid-body algorithms unable to generate correct alignments or retrieve similar structures with large deformations. Therefore, flexibility of proteins should be taken into account when comparing structures and searching for similarities to a query structure.

### Alignment methods

Flexible structure comparison has received much attention in recent years. For instance, FlexProt found congruent rigid fragment pairs between two proteins and the flexible regions (hinges), and then a clustering procedure was performed to join consecutive fragment pairs into congruent domain pairs [19,20]. FATCAT connected aligned fragment pairs based on a dynamic programming algorithm which introduced penalty scores for gaps and twists between consecutive aligned fragment pairs [21]. Compared with FlexProt, FATCAT generates alignments with less twists but similar root mean square deviations (RMSDs) and lengths. The TOPS++FATCAT algorithm reduced the number of aligned fragment pairs during FATCAT comparison processes by applying topological constraints obtained from the alignment of secondary structure elements (SSEs) of TOPS + [22]. Therefore, TOPS++FATCAT is more than 10 times faster compared to FATCAT. Both FlexProt and FATCAT are sequential alignment algorithms thus unable to identify non-sequential alignments. FASE [23] and FlexSnap [24] were designed to tackle the problem of non-sequential flexible structure alignment. FASE compares structures starting from aligned pairs of SSEs with an assumption that an optimal superposition of pairs of structures must have at least one pair of well-aligned SSEs. FlexSnap applies a greedy algorithm for connecting aligned fragment pairs and possesses competitive results against other state-of-the-art pairwise comparison methods. Matt, one of the most popular and accurate flexible multiple structure alignment methods, is also based on the approach of chaining aligned fragment pairs which are allowed translations and rotations during assembling [25,26].

### Non-alignment methods

The alignment/superposition based comparison methods are inefficient for blasting similar structures from a structure database in real-time [27]. Therefore, several non-alignment approaches based on different descriptors of molecular shapes were proposed. These descriptors are usually represented by histograms or vectors, and a similarity score between two molecules is calculated from corresponding descriptors without any alignment [28,29]. For example, Daras *et al.* applied the spherical trace transform method to produce rotational invariant descriptor vectors constituted by weighted geometry- and attribute-based vectors for protein classification [30]. The 3D Zernike descriptor represented a protein structure by 121 numbers based on a series expansion of 3D functions for fast retrieval of similarities, and which demonstrated that low-resolution structures were also applicable [27,31]. Abu Deeb *et al.* proposed a global descriptor on protein surface, and which was constructed from local patch descriptors defined by residue-specific distance distributions between Cα atoms and the numbers of pairwise residue co-occurrences within each surface patch [32]. Yin *et al.* compared local surface of proteins by geometric fingerprints of each surface patch [33]. A fingerprint consists of 60 (4 by 15) bins corresponding to the geodesic-distance-dependent distribution of curvatures.

Nevertheless, most non-alignment methods treated proteins as rigid bodies and neglected flexibility of protein conformations required for performing biological functions. To confront the issue of flexibility, Liu and Fang *et al.* proposed several histogram based descriptors for flexible molecules comparison. For instance, a local diameter descriptor for depicting the local characteristics of boundary points [34], and another descriptor, inner distance, defined as the shortest path between landmark points [28,35]. Both methods are sensitive to self-connection problems during molecular shape deformation. Accordingly, an improved method named Diffusion Distance Shape Descriptor (DDSD) was proposed, which is based on an average distance instead of the shortest distance between two landmark points [36]. Although DDSD is superior to local diameter, inner distance and other descriptors in terms of retrieving similar protein structures, its performance is still unsatisfied with an F_1_-measure of 37.04%.

### Proposed method

Non-alignment or descriptor based approaches are generally fast enough to search a large database in a real-time manner, but do not provide corresponding information of residues which might provide crucial information for biologists. Combining the ideas of alignment and descriptor based approaches, we propose a novel and efficient descriptor called local average distance (LAD) which is based on either geodesic distances (GDs) or Euclidean distances (EDs) for pairwise flexible protein structure comparison. Each protein structure is firstly transformed into its corresponding LAD profile, and the similarity between two proteins is calculated according to pairwise local alignment on transformed profiles. The Hinge Atlas and Hinge Atlas Gold datasets [37] from the MolMovDB [38] were employed to evaluate the performance of proposed LAD descriptors and to compare with several non-alignment and rigid/flexible structure alignment methods.

## Methods

**Figure 1:**
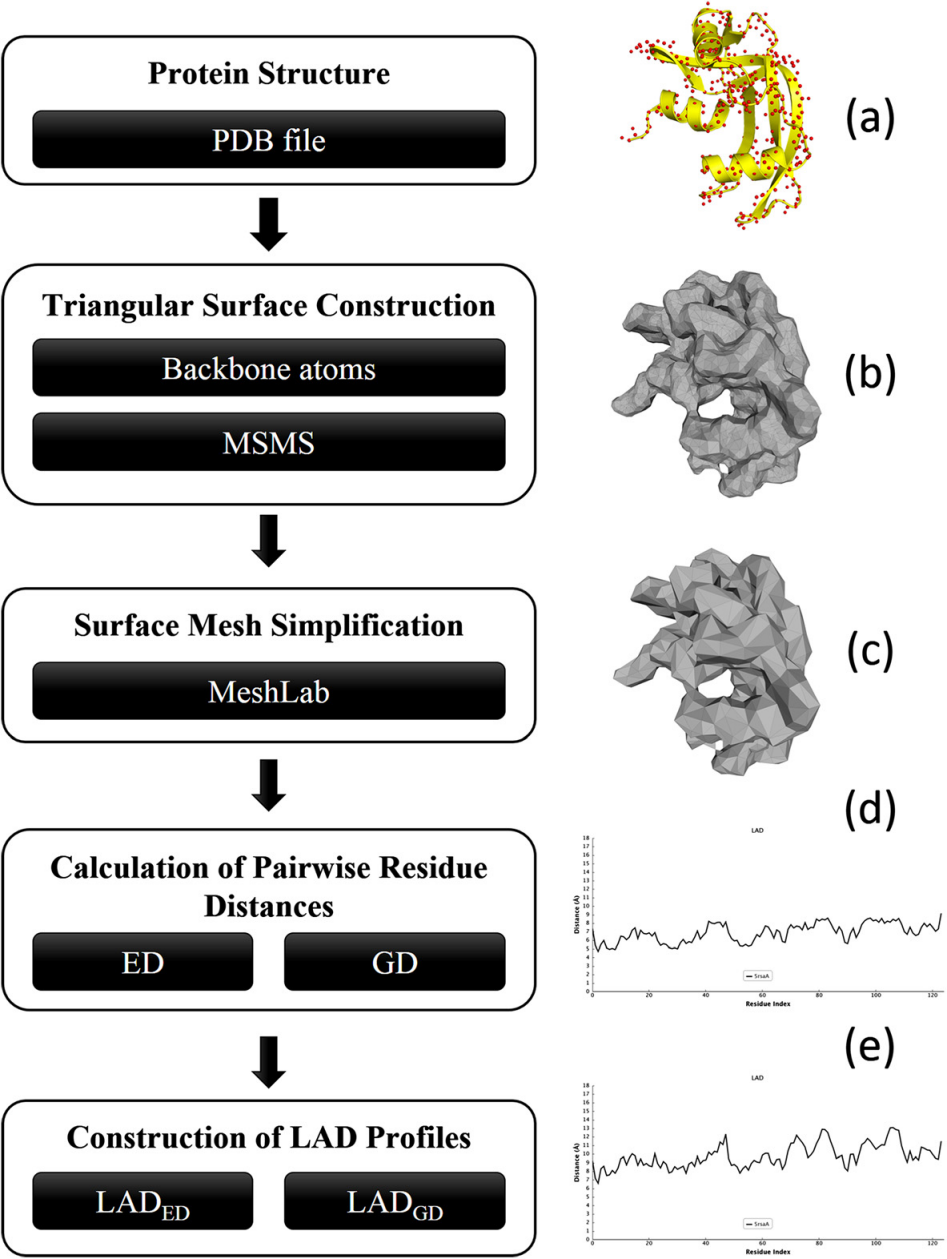
**Flowchart of LAD profile generation for the protein 5rsa:A. (a)** Yellow ribbons display the secondary structures and red spheres represent the backbone atoms (N, Cα, C, O) of each residue. **(b)** The MSMS-generated triangular surface. **(c)** The simplified surface which is created by MeshLab. **(d)** and **(e)** are LAD profiles of the input protein for ED (LAD_ED_) and GD (LAD_GD_) respectively.

The proposed protein structure comparison algorithm is based on the LAD profile which is built from pairwise residue distances (ED or GD) within a protein. The workflow of generating profiles from atomic coordinates of proteins is shown in Figure 1. The similarity between two proteins is determined by a local pairwise alignment of their corresponding LAD profiles. The core procedures can be decomposed into triangular surface construction, surface simplification, ED/GD calculation, profile construction and profile comparison. Details of each step are introduced in the following sections.

### Triangular surface construction and simplification

The solvent-accessible surface (SAS) [39] and solvent-excluded surface [40,41] (SES, also known as molecular surface or Connolly surface) are the most widely used definitions for protein surface analysis. Each atom of a protein is represented as a sphere with its van der Waals radius. The SAS is traced out by the center of a solvent probe sphere rolling over the spherical atoms, whereas the SES is formed by the inward-facing surface of the probe consisting of contact surface and re-entrant surface. For a more complete description of both SAS and SES please refer to [42]. Many algorithms have been developed to build SAS and/or SES such as Gauss-Bonnet theorem [43], level-set [44], alpha shape [45,46], beta shape [47], Euclidean distance transform [48], ray-casting [49]*et al.*[50-52]. One common area-based method defines a residue as a surface residue if its surface area is greater than a specific threshold [46,53]. The other area-based methods consider a residue with relative solvent accessibility larger than a threshold as a surface residue [54,55]. The relative solvent accessibility is defined by taking a residue’s solvent-accessible area divided by the maximum area of that residue [56,57]. In recent years, novel atom-depth-based approaches were proposed as alternative ways to define surface residues [58,59]. Different algorithms employed various definitions of atom depth which could be defined as the distance of an atom from the nearest water molecule surrounding the protein, from the molecular surface, or from its closest solvent-accessible neighbor [60].

The input for building an LAD profile is a standard PDB file. Owing to the requirement of triangular surface meshes for GD calculation, one of the most used and fastest surface program, MSMS v2.6.1 [61], is applied to construct triangular surface meshes from coordinates of all backbone atoms of the protein (Figure 1a). All the parameters of MSMS are remained as default settings. This tool usually generates high resolution meshes (Figure 1b) for proteins. However, it is time-consuming and memory exhausted during the calculation of GDs among mesh vertices. To reduce the resolution of MSMS-generated meshes, an open source tool, MeshLab v1.3.2 (http://meshlab.sourceforge.net/), is adopted to downsample original meshes. The outputs of MSMS are converted into Polygon File Format (Stanford Triangle Format) as MeshLab’s inputs. The algorithm of Quadric edgecollapse, a variant of the well-known quadric error metric algorithm [62] , is employed to simplify meshes (Figure 1c). As a result, the face number of each MSMS-generated mesh could be reduced by 85% generally in this research.

### Calculation of pairwise residue distances

The simplified meshes are then used to identify surface residues, and the GDs and EDs of surface residue pairs can be obtained. Each vertex of a simplified mesh belongs to the closest backbone atom of the protein. In other words, an atom could possess more than one vertex. We defined that the vertices belong to an atom as the associated vertices of that atom. A residue is regarded as a surface residue if its backbone atoms have at least one vertex.

GD is the shortest path along the surface from source to destination points. We adopted the previously published open source program provided by Danil Kirsanov (http://code.google.com/p/geodesic/) to calculate GDs between any two vertices from simplified meshes. The GD between two atoms, *a*_*i*_ and *a*_*j*_ is defined by taking average of GDs from all associated vertices and represented as the following:

$$\mathrm{GD}\left(a_{i},a_{j}\right)=\frac{\sum_{x=1}^{M}\sum_{y=1}^{N}\mathrm{GD}\left(v_{i}^{x},v_{j}^{y}\right)}{M\times N}$$

where *GD*(*a*_*i*_, *a*_*j*_) is the average GD from the *i*^th^ atom to the *j*^th^ atom, $v_{i}^{x}$ and $v_{j}^{y}$ represent the *x*^th^ vertex of the *i*^th^ atom and the *y*^th^ vertex of the *j*^th^ atom respectively. The symbols *M* and *N* indicate the number of vertices associated with the *i*^th^ atom and the *j*^th^ atom, and $\mathrm{GD}\left(v_{i}^{x},v_{j}^{y}\right)$ is the GD from vertex $v_{i}^{x}$ to vertex $v_{j}^{y}$. The atoms possessing no associated vertices won’t be considered, hence *M* and *N* must be strictly larger than zero. In contrast to the measurement of GD, an ED between two atoms can be easily obtained from their coordinates. Once the two different distance measures between any two atoms are obtained, the distance measures between any two residues can be calculated similarly by taking an average of GDs or EDs from all associated backbone atoms.

### Construction of LAD profiles

LAD is proposed to retain local characteristics of each residue in sequential relationship. The LAD profile for a protein consists of average distance values which are built by employing a sliding window scanning from N- to C-terminus. In this study, we have tried different odd window sizes ranging from 3 to 21, and the window size of 9 residues provided the best performance on the training dataset (*Dataset L* from ADiDoS [63]). Hence, a window size of 9 is applied to build all LAD profiles. We have implemented two types of LAD profiles; one is based on ED feature (LAD_ED_, Figure 1d) and the other is based on GD (LAD_GD_, Figure 1e) feature. Given a residue at position *i* (residue_*i*_) in the sequence, the LAD_*i*_ for the residue_*i*_ is defined by taking average distance from residue_*i*_ to both side neighbouring residues within the window.

### LAD diversity

The pairwise structure comparison in this study is based on evaluating the similarities of two LAD profiles from two individual proteins. A variation of Smith-Waterman algorithm is performed to obtain the correspondence of residues between two proteins by comparing LADs instead of amino acid contents. The similarity score between two residues, residue_*i*_ and residue_*j*_, for dynamic programming is inversely proportional to the absolute difference between LAD_*i*_ and LAD_*j*_.

The similarity of two proteins is quantified by the result of pairwise profile alignment. A novel scoring function named as LAD diversity (*LAD*_*div*_) is proposed, which considers the number of equivalent (aligned) residues (*N*_*e*_) and the root-mean-square deviation (RMSD) of LADs for aligned residues. The *LAD*_*div*_ is defined in the following equation where *N*_*Q*_ and *N*_*S*_ are lengths of the query and the subject proteins respectively. The symbols *D* and *α* are used to adjust the effect of RMSD on the *LAD*_*div*_. Since *N*_*e*_ must be less than or equal to *N*_*Q*_ and *N*_*S*_, the value of *LAD*_*div*_ is between 0 and 1, and smaller values represent higher similarities.

$$\mathrm{LA}D_{\mathrm{div}}=1-\frac{N_{e}}{\mathrm{mean}\left(N_{Q},N_{S}\right)\left[1+{\left(\frac{\mathrm{RMSD}}{D}\right)}^{\alpha }\right]}$$

**Figure 2:**
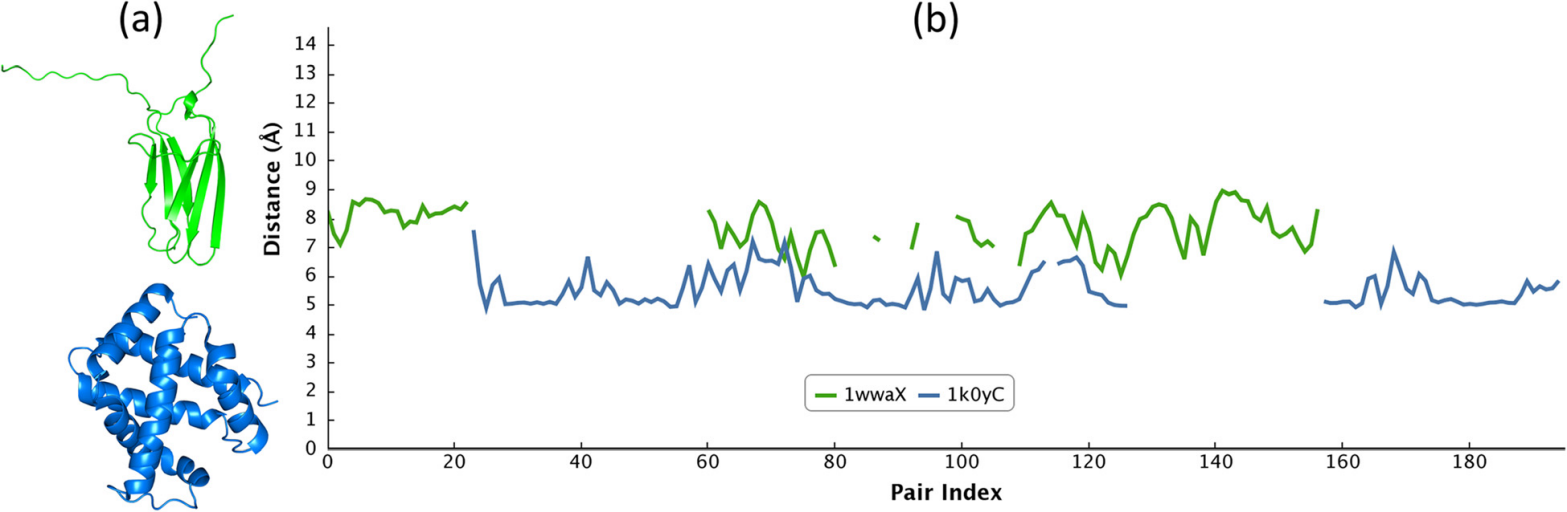
**An example of LAD**_**ED **_**profile alignment for a non-homologous protein pair.** Owing to conformations of both structures are significantly different, their LAD_ED_ profiles could not be aligned properly, and therefore resulted in a high *LAD*_*div*_. **(a)** Cartoon representation for the non-homologous protein pair of 1wwa:X (green) and 1k0y:C (blue). **(b)** The pairwise alignment result of LAD_ED_ profiles for both structures. The x-axis represents the serial numbers of residue pair and the y-axis denotes LAD values.

Profile alignment of a similar structure pair tends to hold a low RMSD and a large *N*_*e*_, and therefore results in a low *LAD*_*div*_. For example, a domain swapping protein pair illustrated in the section of self-connection problem possessing (RMSD, *LAD*_*div*_) of (0.173, 0.0004) and (0.454, 0.02) for LAD_ED_ and LAD_GD_ respectively. Conversely, a dissimilar structure pair possesses a high *LAD*_*div*_ with a large RMSD and a low *N*_*e*_ simultaneously. Figure 2 shows an instance of profile alignment for a non-homologous protein pair which possesses different conformations, and accordingly, the LAD_ED_ profiles obtained high values of (RMSD, *LAD*_*div*_) as (1.601, 0.955) compared to the previous example.

Variables *D* and *α* were trained by the *Dataset L*[63] which contains 706 known domain swapping homologous pairs (*Lds*), 487 common homologous pairs (*Lch*) and 640 non-homologous pairs (*Lnh*) of protein structures. Both *Lds* and *Lch* were considered as a positive dataset in which each pair was anticipated possessing low *LAD*_*div*_ values. Conversely, *Lnh* was considered as a negative dataset which was expected possessing high *LAD*_*div*_ values for each pairs. Let *Lds*_<0.5_ and *Lch*_<0.5_ denote the number of pairs whose *LAD*_*div*_ is less than 0.5 for both *Lds* and *Lch*. The *Lnh*_≥ 0.5_ represents the number of pairs whose *LAD*_*div*_ is larger than or equal to 0.5. We have evaluated *D* ranging from 0.1 to 20 with an interval of 0.1, and a range of 1 to 5 with an interval of 0.5 for *α*. Hence, a total of 1800 (200 × 9) combinations of *D* and *α* were evaluated and the one with maximum *Lds*_<0.5_ + *Lch*_<0.5_ + *Lnh*_≥ 0.5_ was selected. Finally, (*D*, *α*) = (1, 4.5) and (*D*, *α*) = (1.1, 5) were selected for LAD_ED_ and LAD_GD_ respectively.

### Structural diversity

There are many different ways to measure protein structural similarity of aligned results, and many of them have been reviewed in [1]. According to our previous research [63], the structure diversity (*Struct*_*div*_) [64] showed superior performances on distinguishing homologous proteins from non-homologous ones upon various structural comparison methods. Therefore, *Struct*_*div*_ was employed in this study to compare existing rigid/flexible structural alignment tools with our proposed method. *Struct*_*div*_ is defined as:

$$\mathrm{Struc}t_{\mathrm{div}}=\frac{\mathrm{RMSD}}{{\left(\frac{N_{e}}{\mathrm{mean}\left(N_{Q},N_{S}\right)}\right)}^{1.5}}$$

where RMSD is the root mean square deviation of the distances between the aligned Cα atoms. Like *LAD*_*div*_, structural alignment of a similar structure pair tends to have both low RMSD and large *N*_*e*_, and low *Struct*_*div*_.

### Testing datasets

**Figure 3:**
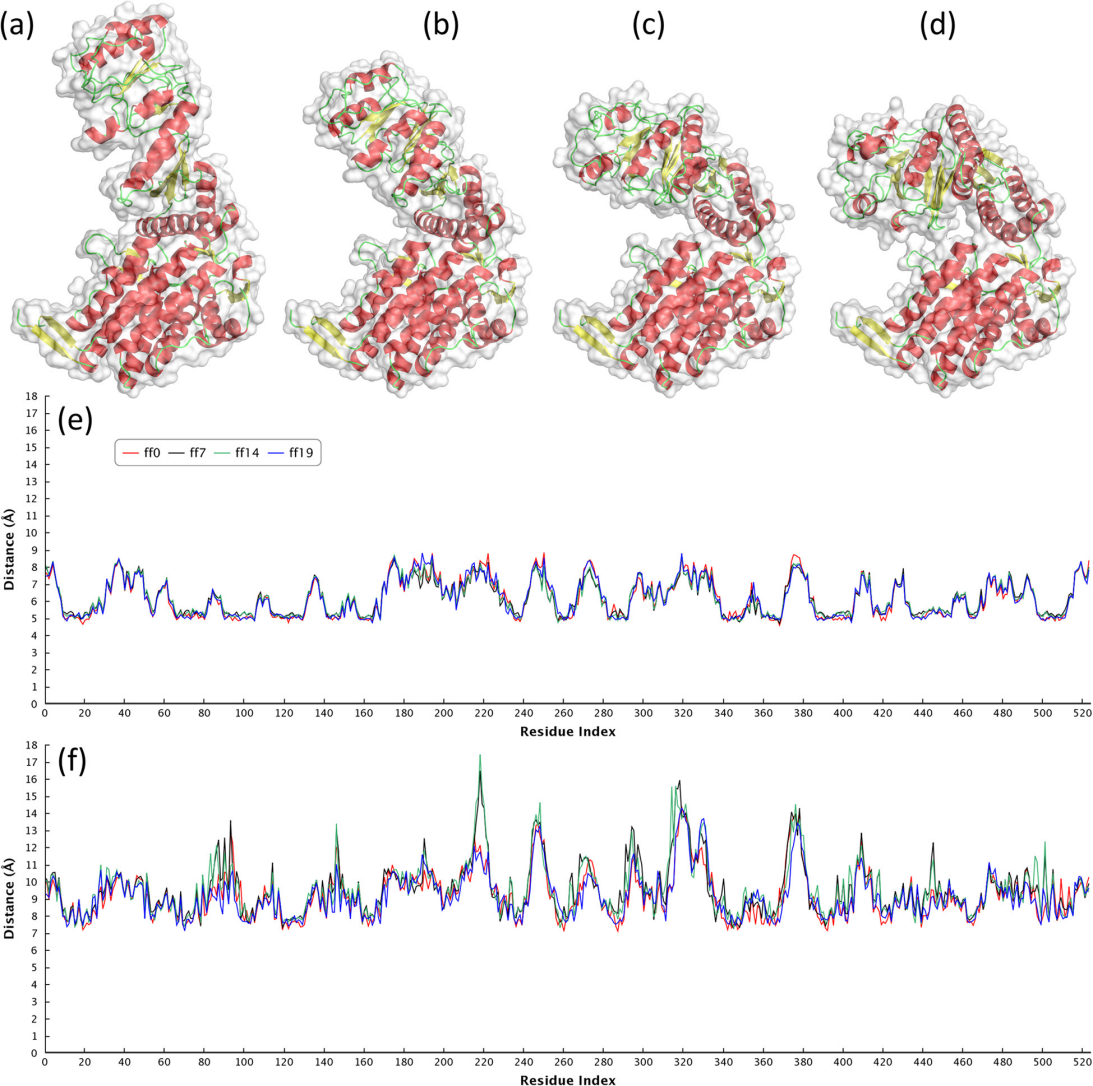
**A morph for the protein GroEL in the Hinge Atlas dataset.** There are 20 structures in this morph (morph id is 805511–5128) containing 4 hinge residues: 191G, 192 M, 372A and 373G. **(a)** and **(d)** are the first and last proteins in the morphing group respectively. **(b)** is the 7^th^ interpolated structure and **(c)** for the 14^th^ structure. **(e)** and **(f)** represent the LAD_ED_ and LAD_GD_ profiles for the four structures of the same protein and both profiles are insensitive to the conformational changes. The x-axis represents the serial numbers of residues and the y-axis denotes LAD values. Figures **(a)-(d)** were generated by PyMOL (http://www.pymol.org/), and **(e)-(f)** by Highcharts (http://www.highcharts.com/).

There were two testing datasets applied in this research to validate our method and compare with existing methods. The first one is Hinge Atlas dataset which contains 2791 protein structures of 214 non-redundant morphs exhibiting hinge bending motions. The lengths of proteins range from 28 to 994 residues. A morph is a group of structures (9 to 32) comprising two homologous proteins with different conformations and several interpolated structures between these two initial structures. About 97% of morphs in the dataset possess three or less hinge points. Figure 3 shows an example of morph with a large conformational change for the protein GroEL containing 524 residues. Neither LAD_ED_ and LAD_GD_ descriptors are sensitive to the deformation, especially for LAD_ED_. The second dataset provided by Liu *et al.* was a subset of Hinge Atlas [37] and Hinge Atlas Gold datasets, and which was applied in the previous study [36]. The Liu’s dataset contains 382 protein structures of 27 groups with large degrees of conformational changes.

## Results

### Comparison with structural alignment methods

**Figure 4:**
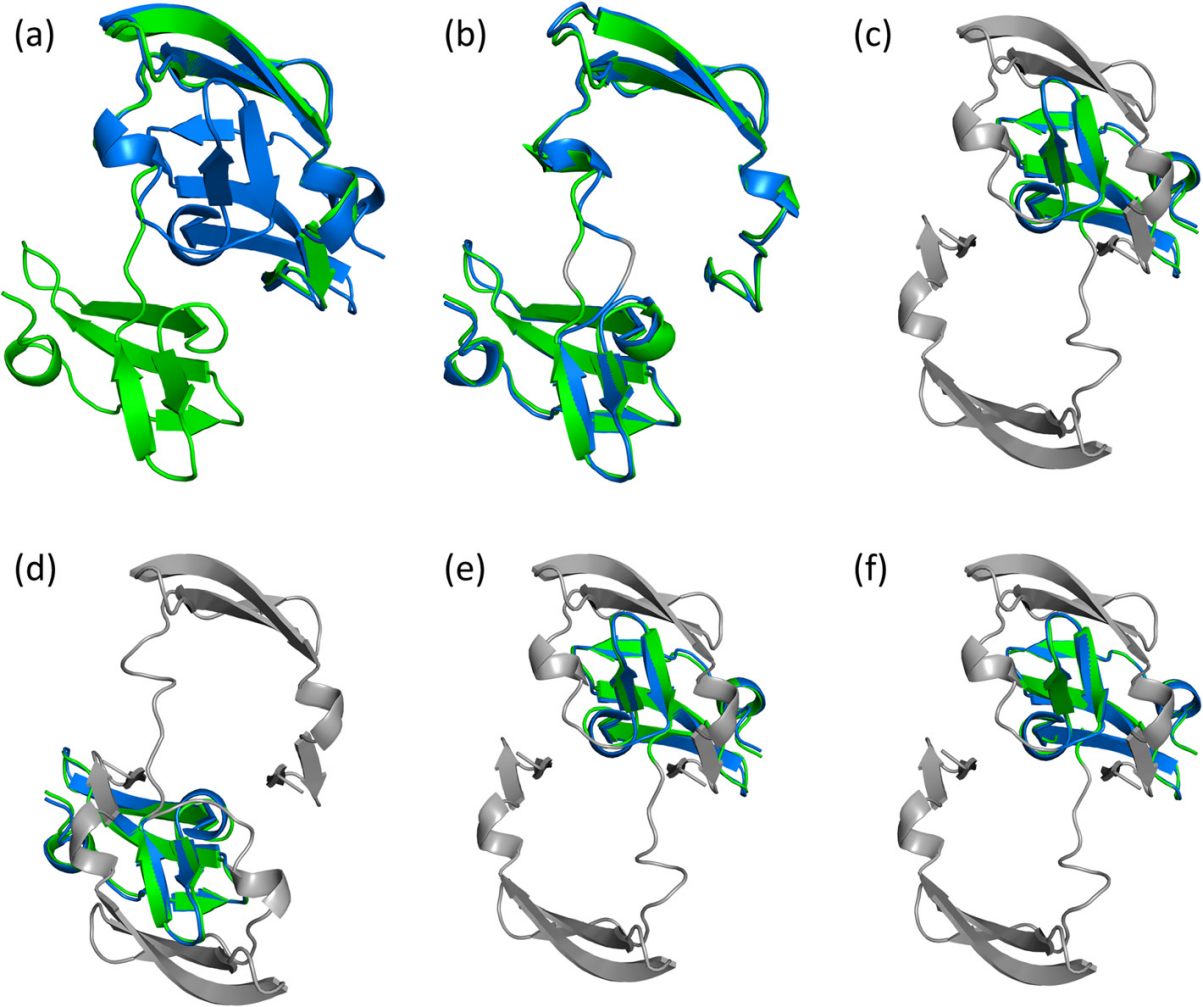
**An example of successful retrieval but with poor structure alignments.** The structure pair is from the morphing group of va2eznA-1l5bA in the Hinge Atlas dataset. **(a)** The open-form (green, ff9) and closed-form (blue, ff0) of Cyanovirin-N. **(b)** to **(f)** are structure alignments generated by jFATCAT, FASE, Fast, Matt-Rigid and Matt-Flexible respectively. The non-aligned regions are colored by gray. All methods ranked the closed-form of Cyanovirin-N at the top of 214 representative structures when the open-form of Cyanovirin-N as a query; nevertheless, FASE, Fast, Matt-Rigid and Matt-Flexible only aligned half portion of the query protein.

LAD descriptors were compared with 2 rigid and 5 flexible structural alignment methods on the Hinge Atlas dataset in terms of retrieving similar structures which belong to the same group (morph) as the query structure. The first structure in each group was regarded as the representative for that group, and the remaining 2577 proteins were considered as query structures. Each query protein compared with 214 representatives, and there were a total of 551478 (2577 x 214) pairwise comparisons. The results for each query were sorted according to the diversity scores (*LAD*_*div*_ or *Struct*_*div*_), and it was regarded as a successful retrieval if the representative belonging to the same group as query proteins was ranked at the first place. The retrieval performance for LAD and other structural alignment methods on the Hinge Atlas dataset were summarized in Table 1. The results have shown that LAD_ED_ and LAD_GD_ performed better than other methods and achieved retrieval success rates of 97.1% and 95% respectively. The structural alignment methods generated unsatisfied alignment results even though the relevant structures were successfully retrieved at the first place. For example, all methods ranked the relevant structure of ff0 at the top position for the query structure of ff9 from the morph group of va2eznA-1l5bA, and it is a domain-swapped dimer of Cyanovirin-N (Figure 4a). In this case, LAD_ED_, LAD_GD_, FlexProt, FlexSnap and jFATCAT (Figure 4b) could align the protein pair completely, but FASE (Figure 4c), Fast (Figure 4d), Matt-Rigid (Figure 4e) and Matt-Flexible (Figure 4f) only aligned half portion of the structure.

**Table 1:** Retrieval performances of 2577 queries for different methods on the Hinge Atlas dataset

**Method**	**Number of successful retrieval**	**Success rate (%)**
LAD_ED_	2502	97.1
LAD_GD_	2447	95.0
Matt-Flexible	2342	90.9
FlexSnap	2329	90.4
FASE	2282	88.6
jFATCAT	2241	87.0
FAST^*^	2234	86.7
Matt-Rigid^*^	2185	84.8
FlexProt	2167	84.1

^*^Rigid alignment method.

The results are ordered by the success rates and show that both LAD_ED_ and LAD_GD_ outperform other methods.

In addition to the measure of successful retrieval rates, we also evaluated the performances for the Hinge Atlas dataset based on the precision-recall curve of 11-point interpolated average precision which is a common measurement in information retrieval systems [65]. It should be noted that the 214 representatives were treated as query structures individually, and each of them compared with the remaining 2577 structures in order to search structures belonging to the same group. A precision rate is the fraction of retrieved structures that are relevant to the query protein, and a recall rate is the fraction of relevant structures that are successfully retrieved. Precision and recall rates are defined in the following equations:

$$\mathrm{Precision}=\frac{\mathrm{TP}}{\mathrm{TP}+\mathrm{FP}}$$

$$\mathrm{Recall}=\frac{\mathrm{TP}}{\mathrm{TP}+\mathrm{FN}}$$

**Figure 5:**
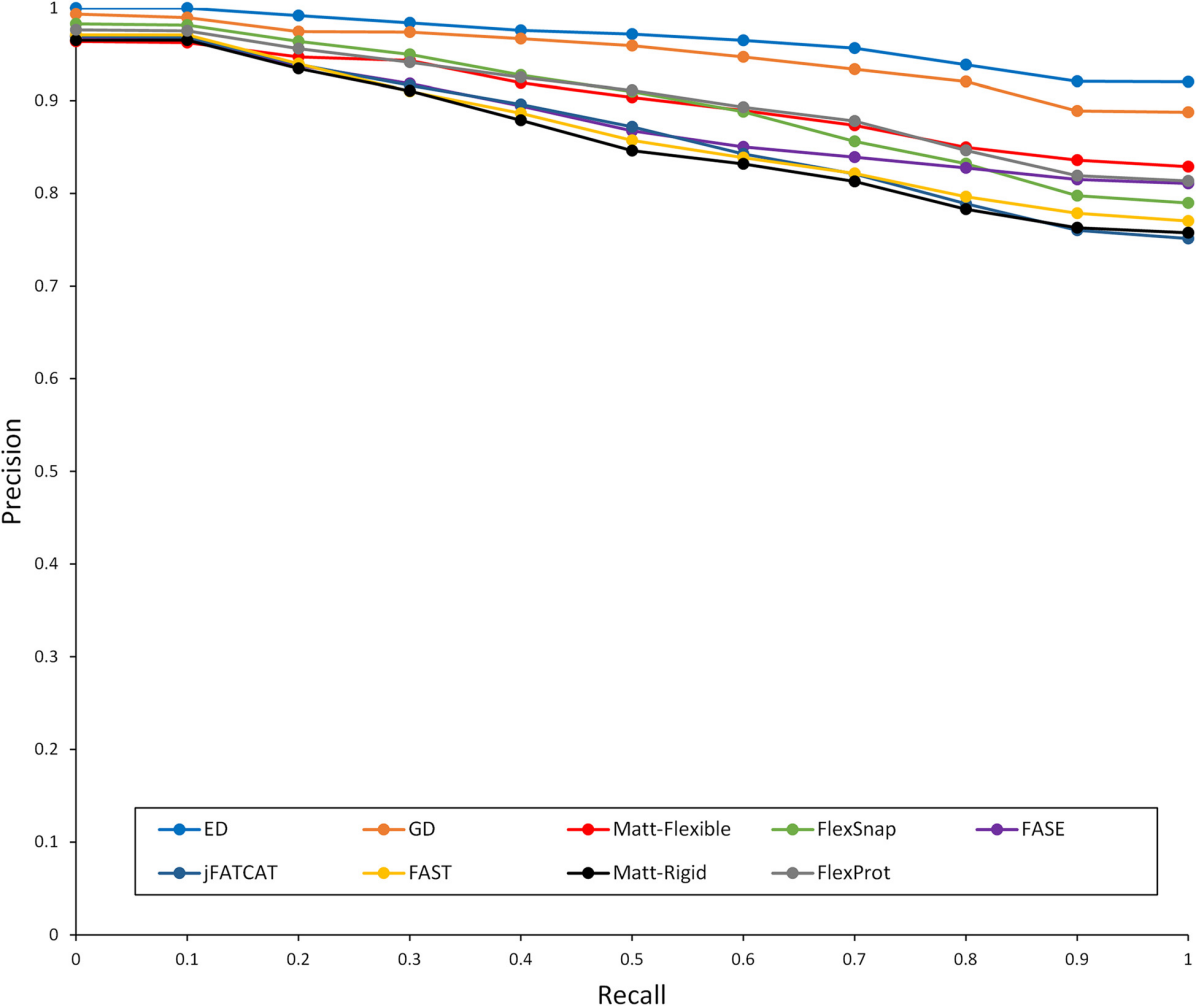
**Precision-recall curves of 11-point interpolated average precision for different methods on the Hinge Atlas dataset.** The top blue and orange curves represent LAD_ED_ and LAD_GD_ respectively, and show that both LAD methods provide the best performance.

True positive (*TP*) is the number of successful retrieved structures; false positive (*FP*) represents the number of inaccurately retrieved structures; false negative (*FN*) denotes the number of structures belonging to the same group as query but not being retrieved. The interpolated precision for a specific recall *r* is defined as the maximum precision over any recall *r* ' ≥ *r*[65]. For each query, a set of 11 interpolated precisions at 11 recall levels (0, 0.1, 0.2 … 1) were determined, then averages of interpolated precisions for 214 queries at each level were calculated. According to the precision-recall curves (see Figure 5), both LAD_ED_ and LAD_GD_ outperformed other methods since they possessed larger area under the curve.

*R*-Precision and Mean Average Precision (MAP) are the other common quantitative measures for evaluating overall performance of information retrieval systems. If there are total *R* relevant structures for a query, *R*-Precision is defined as the number of relevant structures in the top *R* retrieved structures divided by *R*. For a query, Average Precision is an average of precisions for each relevant structure. MAP is defined as the mean of the Average Precisions for a set of queries. For more details of calculating these measures please refer to [65]. The average *R*-Precision and MAP of 214 queries for different methods are shown in Table 2. The results have shown that both LAD_ED_ and LAD_GD_ performed superior to other methods, and LAD_ED_ achieves an average of 95.54% for *R*-Precision and 96.67% for MAP.

**Table 2:** Retrieval performances of 214 queries for different methods on the Hinge Atlas dataset

**Method**	**Average *R*-precision (%)**	**Mean average precision (%)**
LAD_ED_	95.54	96.67
LAD_GD_	93.53	94.95
Matt-Flexible	87.55	89.62
FlexSnap	86.97	89.71
FASE	84.97	87.40
jFATCAT	83.36	86.23
FAST	82.81	86.16
Matt-Rigid	82.24	85.33
FlexProt	87.14	89.98

### Comparison with non-alignment methods

The Liu’s dataset was employed to compare LAD descriptor with non-alignment methods. In order to compare with the results in [36], only the top 64 retrieved structures for each query were used to compute the precision and recall rates. The F_1_-measure is the harmonic mean of recall and precision rates defined as:

$$F_{1}-\mathrm{measure}=\frac{2\times \mathrm{Precision}\times \mathrm{Recall}}{\mathrm{Precision}+\mathrm{Recall}}$$

where the maximum value is 1. In contrast to the arithmetic mean, both precision and recall rates need to be high to obtain a high F_1_-measure. The retrieval performance of F_1_-measure is listed in Table 3. LAD_ED_ and LAD_GD_ achieved 43.27% and 43.18% of F_1_-measure respectively and outperformed the other 7 non-alignment methods with a highest F_1_-measure of 37.04%.

**Table 3:** Comparison with non-alignment methods on Liu’s dataset

**Method**	**F**_**1**_**-measure (%)**
LAD_ED_	43.27
LAD_GD_	43.18
Diffusion distance (DD)	37.04
Inner distance (ID)	35.83
Shape distribution (SD)	28.40
Euclidean distance (ED)	28.81
Solid angle histogram (SAH)	25.69
Geodesic distance (GD)	26.42
Spherical harmonic descriptor (SHD)	23.93

The results are taken from [36] except LAD_ED_ and LAD_GD_.

## Discussion

### Self-connection problem

**Figure 6:**
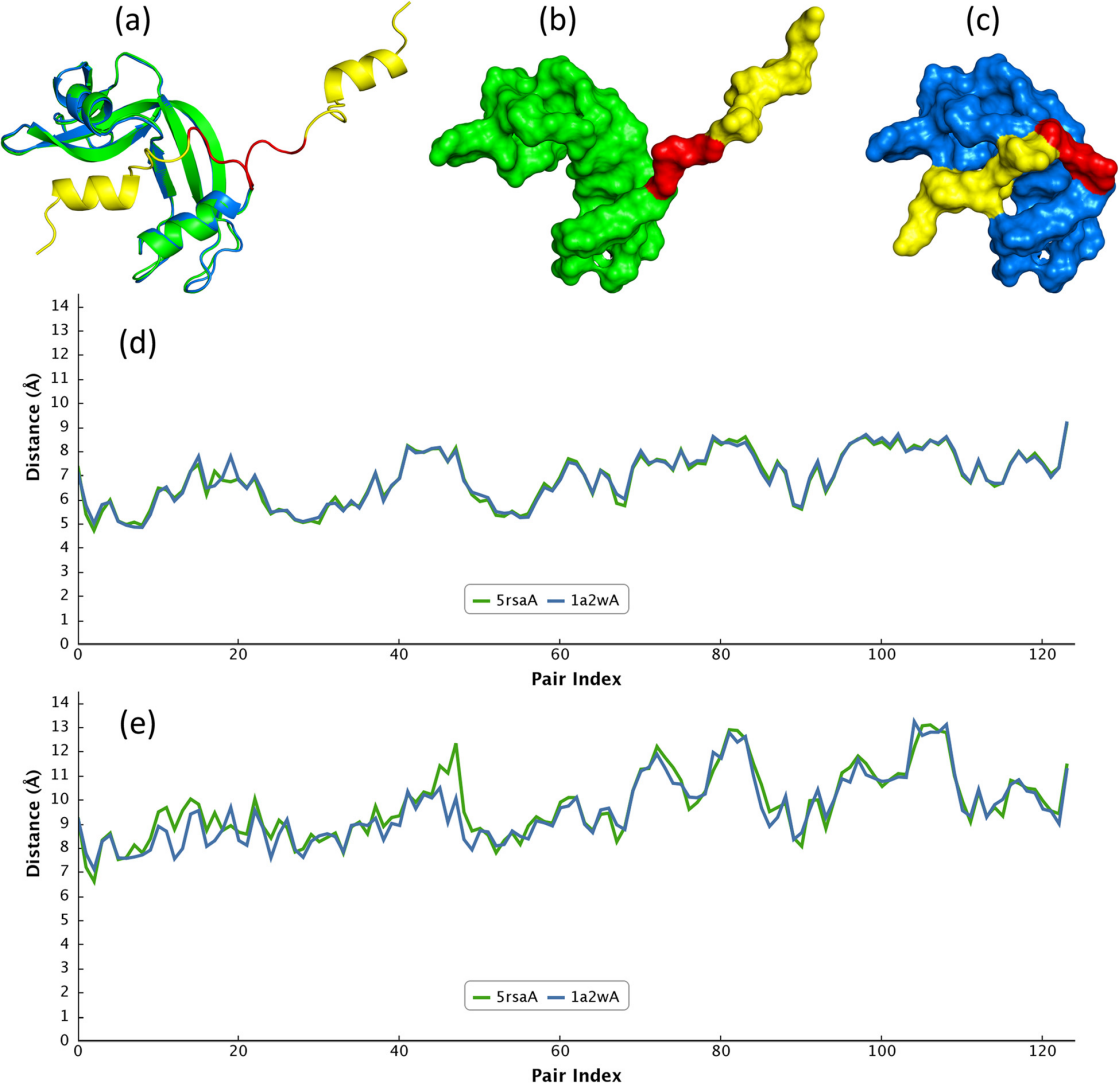
**An example of self-connection case for domain swapping proteins of 1a2w:A and 5rsa:A.** The yellow part of open-form Ribonuclease A (PDB code: 1a2w, chain A) swaps toward the protein body to form a closed-form (PDB code: 5rsa, chain A). **(a)** The structural alignment of 1a2w:A (green) and 5rsa:A (blue); the hinge loops are highlighted in red. The backbone surfaces of 1a2w: A **(b)** and 5rsa:A **(c)** are different due to domain swapped and self-connected formation. However, LAD_ED_**(d)** and LAD_GD_**(e)** profiles for both structures remain consistent.

Figure 6 is an example of bona fide domain swapping protein pair holding self-connection on surface caused by a large hinge bending motion. The difficulty is that a self-connection leads to topology changes, hence the inner distance method considering all landmark points cannot solve this problem [35,36]. However, this type of deformation can be overcome by our proposed descriptor especially for LAD_ED_ approach since an LAD only considers the local geometric properties which are not sensitive to global topology changes. Figure 6d and Figure 6e have shown a high consistency of LAD_ED_ and LAD_GD_ profiles between open-form (PDB code: 1a2w, chain A) and close-form (PDB code: 5rsa, chain A) of Ribonuclease A respectively. It is obvious that LAD_ED_ is more consistent than LAD_GD_ in this case, but both *LAD*_*div*_ are close to zero representing highly similar conformations. The (RMSD, *LAD*_*div*_) for LAD_ED_ is (0.173, 0.0004) and (0.454, 0.02) for LAD_GD_.

**Figure 7:**
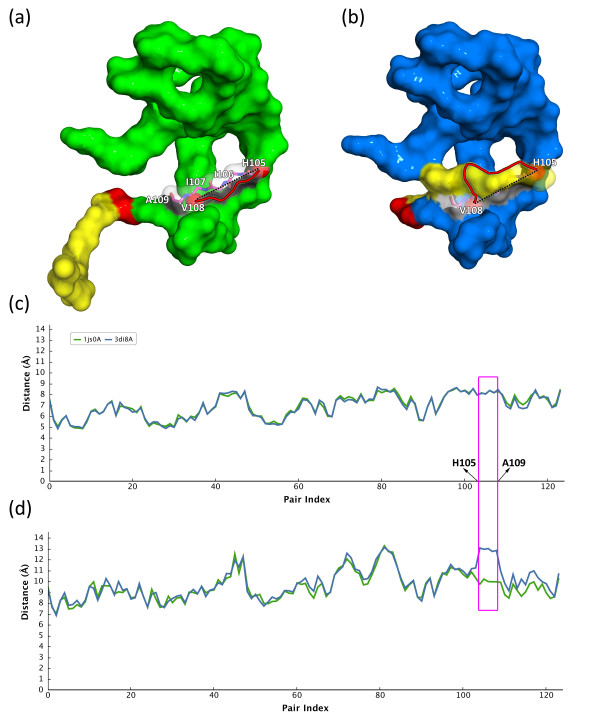
**Illustrating the variation between LAD**_**ED **_**and LAD**_**GD **_**for a self-connection case.**The 3D domain-swapped Ribonuclease A consists of a protein body (green/blue surface), a hinge loop (red surface) and a swapped domain (yellow surface). **(a)** The open-form Ribonuclease A (PDB code: 1js0, chain A) **(b)** The domain-swapped closed homolog of **(a)** (PDB code: 3di8, chain A). **(c)** and **(d)** are LAD_ED_ and LAD_GD_ profiles of both closed- and open-form structures respectively. The red solid curve of **(a)** and **(b)** denotes a GD path, which is the shortest path along the surface (white surface region) connecting two residues H105 and V108 (red spheres). The residues from H105 to A109 of both proteins are shown as magenta sticks and highlighted within a magenta box in **(c)** and **(d)**. The black dashed line of **(a)** and **(b)** indicates the ED path between the residues H105 and V108. Note that the magenta box has shown that the LAD_GD_ profile is more sensitive at the topological changed locations than the LAD_ED_ profile.

In general, LAD descriptors are insensitive to self-connection cases; however, an LAD_GD_ profile is sometimes not consistent at the location of self-connecting regions. Given another domain swapping example in Figure 6, an open-form Ribonuclease A (PDB code: 1js0, chain A) changes to a closed-form (PDB code: 3di8, chain A). The swapped domain (yellow surface) bends and intertwines with the protein body (blue surface) via conformational changes of highly flexible hinge loops (red surface) (see Figure 7a and Figure 7b). In Figure 7c, it is obvious that the LAD_ED_ varies slightly between the open- and close-form states from H105 to A109 residues (magenta rectangle). In contrast, the LAD_GD_ of close-form state is higher than that of open-form state at corresponding highlighted regions (see Figure 7d). For a detailed illustration, it can be imagined a path from the residue H105 to its +3 position (V108). When the swapped domain locates apart from the protein body in the open-form state, the GD between these two residues is the shortest path along the white surface. The GD and ED between the two residues in the open-form state are 11.12 Å and 10.37 Å respectively. However, the path was changed while the swapped domain bending to the body and intertwining with the white surface region forming a self-connection case. The GD is increased significantly due to an additional mountain (yellow region in Figure 7b) obstructing the original path from residue H105 to V108. The ED maintained high similarity since its path directly passed through the mountain instead of along on the surface. The GD and ED between the two residues of the close-form state are 16.77 Å and 9.57 respectively. This phenomenon is the main reason why an LAD_GD_ descriptor more sensitive to the topological changes than LAD_ED_.

### Differences between the previous and proposed ED/GD based methods

In previous studies [34-36], ED and GD were shown to be sensitive to shape deformation and not feasible for flexible molecular shape comparison. However, it is interesting that relying on the proposed LAD methods, both features become insensitive to topological changes and reveal deformation invariant properties to tackle with the flexibility problems. The reason for sensitive ED and GD features in previous studies is that both distances were computed among all global landmark points. On the contrary, the LAD exploits the characterization of local geometric features for each residue and its neighbouring residues. Therefore, ED and GD features become much less sensitive to global topological changes.

### Computational time

Pairwise comparison of LAD profiles was performed by a modification of Smith-Waterman algorithm and possessed the same time complexity. The goal of a sequence alignment problem is to identify the correspondence of residues between two given proteins, while a structure alignment emphasizes on finding both an alignment and a spatial superposition. Possible combinations of corresponding residues are countable while possibilities of special superposition are innumerable. Therefore, the computational complexity of the proposed algorithm is inherently less than most commonly used structure alignment methods [66]. The LAD algorithm was implemented by C# .NET running on an Intel Core i5-2500 3.3GHz computer with 16GB ram. According to the 551478 pairwise comparisons mentioned in the result section, it only cost an average computational time of 3.896 and 4.828 milliseconds per comparison for LAD_ED_ and LAD_GD_ profiles respectively.

## Conclusions

We proposed a novel profile-based alignment method, named LAD, for pairwise flexible protein structure comparison. It can be constructed in a sense of any kind of spatial measures of local neighbouring residues within a specific sliding window. Here, GD and ED were used to build LAD_GD_ and LAD_ED_ profiles. The idea of LAD improves the ED- and GD-based descriptors which were previously shown to be sensitive to molecular shape deformation, in particular to topologically structural changes. The effectiveness of LAD descriptor has been evaluated on two datasets of hinge bending motions from the MolMovDB. Our methods are robust to deformed flexible molecules and achieve good performance regarding assignment of the queries to different classes of molecules with conformational changes, and the results have shown superior performance compared to existing alignment- and nonalignment-based tools. Finally, the reasons of LAD descriptor being insensitive to flexible proteins with self-connection circumstance was described by taking 3D domain swapping cases as examples, and further discussion of LAD_ED_ possessing more robust properties than LAD_GD_ was also explained. Required computational time for pairwise LAD_ED_/LAD_GD_ profile comparisons was analyzed to demonstrate its feasibility for constructing an on-line structure comparison system. The proposed descriptor is indeed effective in retrieving deformed proteins and it could be an alternative approach for database search, discovery of previously unknown conformational relationships, and reorganization of protein structure classification.

### Availability of supporting data

The training and testing datasets for our method can be obtained from previously published papers by Chu CH [63] and Flores SC [37,38].

## Abbreviations

LAD: Local average distance; ED: Euclidean distance; GD: Geodesic distance; MAP: Mean average precision.

## Competing interests

The authors declare that they have no competing interests.

## Authors’ contributions

HWW, CHC and TWP conceived the algorithm. HWW and CHC implemented the algorithm, performed the experiments and wrote the manuscript. TWP and WCW proofread and revised the manuscript. All authors read and approved the final manuscript.
